# Decompressive craniectomy in trauma: What you need to know

**DOI:** 10.1097/TA.0000000000004357

**Published:** 2024-08-09

**Authors:** Georgios Solomou, Jesvin Sunny, Midhun Mohan, Iftakher Hossain, Angelos G. Kolias, Peter J. Hutchinson

**Affiliations:** From the Division of Neurosurgery, Department of Clinical Neurosciences (G.S., J.S., M.M., I.H., A.G.K. P.J.H.), Addenbrooke's Hospital, University of Cambridge, Cambridge; National Hospital for Neurology and Neurosurgery (J.S.), London, United Kingdom; and Neurocenter (I.H.), Department of Neurosurgery, Turku University Hospital, Turku, Finland.

## Abstract

Decompressive craniectomy (DC) is a surgical procedure in which a large section of the skull is removed, and the underlying dura mater is opened widely. After evacuating a traumatic acute subdural hematoma, a primary DC is typically performed if the brain is bulging or if brain swelling is expected over the next several days. However, a recent randomized trial found similar 12-month outcomes when primary DC was compared with craniotomy for acute subdural hematoma. Secondary removal of the bone flap was performed in 9% of the craniotomy group, but more wound complications occurred in the craniectomy group. Two further multicenter trials found that, whereas early neuroprotective bifrontal DC for mild to moderate intracranial hypertension is not superior to medical management, DC as a last-tier therapy for refractory intracranial hypertension leads to reduced mortality. Patients undergoing secondary last-tier DC are more likely to improve over time than those in the standard medical management group. The overall conclusion from the most up-to-date evidence is that secondary DC has a role in the management of intracranial hypertension following traumatic brain injury but is not a panacea. Therefore, the decision to offer this operation should be made on a case-by-case basis. Following DC, cranioplasty is warranted but not always feasible, especially in low- and middle-income countries. Consequently, a decompressive craniotomy, where the bone flap is allowed to “hinge” or “float,” is sometimes used. Decompressive craniotomy is also an option in a subgroup of traumatic brain injury patients undergoing primary surgical evacuation when the brain is neither bulging nor relaxed. However, a high-quality randomized controlled trial is needed to delineate the specific indications and the type of decompressive craniotomy in appropriate patients.

## THE EPIDEMIOLOGY AND ECONOMIC COST

Traumatic brain injury (TBI) is a leading cause of injury-related death and disability, with an annual incidence estimated at 27 to 69 million and $600,000 to $1.8 million lifetime cost per case.^1,2^ Roughly 55 million people (prevalence), corresponding to 0.7% of the world's population, suffer from a TBI globally.^3^ A recent systematic review in Europe estimated the crude incidence to range from 47.3 to 848 per 100,000, with the crude mortality ranging from 3.3 to 28.1 per 100,000.^3^ However, global estimates denote that low- and middle-income countries (LMICs) are disproportionally affected, with three times more TBI cases than high-income countries.^1,4^ Males can be affected twice as much as females.^5^ The distribution of TBI across ages is bimodal, highest among the pediatric population and older adults.^5^ Falls and motor-vehicle crashes predominate in TBI-related deaths in civilians, while blast-induced TBI is the most frequent type in the military.^1–4^ In high-income countries, TBI in the elderly is increasing because of falls, whereas in LMICs, TBI due to road traffic accidents predominates.^1–5^ Traumatic brain injury is a significant global public health issue.

## MEDICAL AND INTENSIVE CARE MANAGEMENT

**Figure 1:**
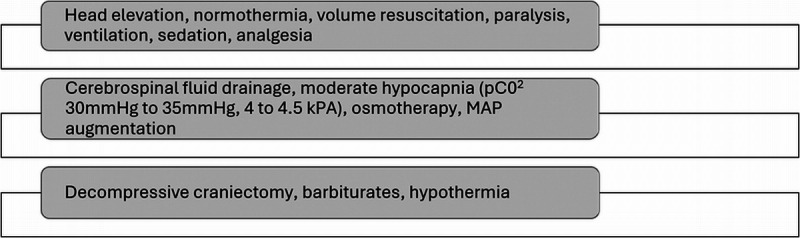
Summary of treatments for raised ICP due to TBI.

After TBI, there may be a rise in intracranial pressure (ICP) owing to the mass effect from hematomas, contusions, and diffuse and localized swelling.^6,7^ A rise in ICP leads to a reduction in cerebral perfusion pressure, resulting in ischemia and, subsequently, cell death associated with increased mortality.^8^ The Brain Trauma Foundation (BTF) guidelines (Fourth Edition) recommend the management of severe TBI using ICP monitoring data to reduce in-hospital and 2-week postinjury mortality.^9^ The treatments for raised ICP due to TBI are briefly summarized in Figure 1.

## SURGICAL MANAGEMENT FOR TBI

Decompressive craniectomy (DC) is a neurosurgical procedure in which a large section of the skull is removed, and the underlying dura mater is opened, leading to a reduction in ICP and alleviating cerebral hypoperfusion. Primary DC refers to leaving a large part of the skull (bone flap) out after evacuating an intracranial mass lesion, such as an extradural, subdural, or intraparenchymal traumatic hematoma or a cerebral contusion, during the early phase of TBI.^10–12^ Primary DC is most frequently performed following evacuation of an acute SDH because these lesions are often associated with parenchymal injuries and swelling. On the other hand, when ICP remains refractory to treatments offered as part of a tiered protocol, secondary DC can be offered as a third-tier therapy.^10–12^ A secondary DC can be bifrontal (bone flap extends from the floor of the anterior cranial fossa anteriorly to the coronal suture posteriorly and to the middle cranial fossa floor bilaterally), unilateral, or bilateral.

## INDICATIONS

Primary DC has not been studied extensively until recently.^13^ The Milan Consensus Conference on Clinical Applications of Intracranial Pressure Monitoring in Traumatic Brain Injury in 2014 concluded that there is a low risk of raised ICP after epidural hematoma evacuation, suggesting that DC is not routinely required for treatment of isolated epidural hematoma.^6,14^ Two thirds of TBI patients undergoing surgery (excluding external ventricular drain or ICP monitoring insertion) have an evacuation of an acute subdural hematoma (ASDH), which is associated with a high mortality rate.^15,16^ Acute subdural hematoma is often associated with the presence of intraparenchymal contusions or hematomas, as well as brain swelling.^15,16^ Ample evidence indicates that a significant proportion of patients develop intracranial hypertension postoperatively after surgery for ASDH, and this is associated with a high mortality rate.^15,16^ The BTF guidelines recommend performing surgical evacuation with a craniotomy with or without replacement of the bone flap but do not specify the exact indications for DC.^9,17^ Moreover, significant heterogeneity of practice has been reported among neurosurgeons in choosing primary DC over craniotomy.^18,19^ Some studies suggest better postoperative ICP control with DC than craniotomy.^20,21^ However, comparative effectiveness studies sometimes lead to controversial conclusions regarding mortality and functional outcomes.^22,23^

## PRIMARY DC

In 2023, results of the Randomized Evaluation of Surgery with Craniectomy for Patients Undergoing Evacuation of Acute Subdural Hematoma (RESCUE-ASDH) were published.^13^ The RESCUE-ASDH was a multicenter, pragmatic, parallel-group randomized trial comparing the clinical efficacy and cost-effectiveness of primary DC versus craniotomy in adult head-injured patients undergoing evacuation of an ASDH.^13^ The trial inclusion criteria are outlined in Table 1. Patients were excluded if they had bilateral ASDHs, which both required evacuation, severe preexisting physical or mental disability, or comorbidity. Eligible patients were randomized intraoperatively after the ASDH was evacuated. Patients with significant brain swelling preventing safe replacement of the bone flap were not suitable for randomization and were followed up in an observational cohort. The primary outcome measure was the Glasgow Outcome Scale—Extended (GOSE) at 12 months postinjury. Outcome analyses were performed in the modified intention-to-treat population, which included all randomly assigned patients except those who withdrew consent for participation in the trial and those who were lost to follow-up.

**TABLE 1:** Summary of Inclusion Criteria, Patient Groups, and Outcomes From the RESCUE-ASDH, RESCUEicp, and DECRA Trials

	DECRA^12^		RESCUEicp^24^		RESCUE-ASDH^13^	
Inclusion criteria	Patients 15 to 59 y of age with severe TBI		Patients 10 to 65 y of age, with TBI and refractory intracranial hypertension (>25 mm Hg)		Patients older than 16 y and have an ASDH that warranted evacuation with DC or craniotomy (with bone flap at least 11 cm in both instances)	
Timing of randomization	After tier 1 therapies		After tiers 1 and 2 therapies		At the same time as evacuation of ASDH	
ICP threshold for randomization	>20 mm Hg for 15 min in 1 h		>25 mm Hg for at least 1 h		N/A	
Primary outcomes	GOSE at 6 mo		GOSE at 6 mo		GOSE at 12 mo	
Treatment	DC	Medical therapy	DC	Medical therapy	DC	Craniotomy
No. patients	73	82	202	196	222	228
Mean age, y	23.7	24.6	32.3	34.8	48.8	48.3
Description	Large bifronto-temporo-parietal craniectomy with bilateral dural opening	Ongoing medical therapy	Large unilateral fronto-temporo-parietal craniectomy or bifrontal craniectomy	Ongoing medical therapy with the option of adding barbiturates	The dura was left open, or a noncontracting duroplasty was performed, and the bone flap was left out	The bone flap was replaced and fixed to the surrounding skull before scalp closure
GOSE score at 6 mo	*Favorable: 32 (30%) Unfavorable: 51 (70%)	*Favorable: 40 (49%) Unfavorable: 42 (51%)	**Favorable: 86 (42.8%) Unfavorable: 115 (57.2%)	**Favorable: 65 (34.6%) Unfavorable: 123 (65.4%)	**Favorable: 85 (42%) Unfavorable: 116 (58%)	**Favorable: 102 (49%) Unfavorable: 104 (51%)
GOSE score at 12 mo	*Favorable: 30 (41%) Unfavorable: 43 (59%)	*Favorable: 43 (52%) Unfavorable: 39 (48%)	**Favorable: 88 (45.4%) Unfavorable: 106 (54.6%)	**Favorable: 58 (32.4%) Unfavorable: 121 (67.6%)	**Favorable: 96 (46%) Unfavorable: 115 (54%)	**Favorable: 107 (50%) Unfavorable: 108 (50%)
GOSE at 24 mo	N/A	N/A	**Favorable: 82 (45.1%) Unfavorable: 100 (54.9%)	**Favorable: 54 (31%) Unfavorable: 120 (69%)	N/A	N/A
ICP after intervention, mm Hg	14.4 ± 6.8	19.1 ± 8.9	Median: 14.5 (IQR, 1.7–18)	Median: 17.1 (IQR, 4.2–21.8)	N/A	N/A

*Favorable outcomes were defined as lower moderate disability and above. **Favorable outcomes were defined as upper severe disability or better on the GOSE. IQR, interquartile range; N/A, not applicable.

In the modified intention-to-treat ordinal analysis of GOSE ratings at 12 months, the common odds ratio across outcome categories for the craniotomy group compared with the DC group was 0.85 (95% confidence interval [CI], 0.60–1.18; *p* = 0.32). In the prespecified secondary fixed-dichotomy analysis, unfavorable outcomes at 12 months (defined as death, vegetative state, or lower severe disability on the GOSE) were reported in 50.2% in the craniotomy group and 54.5% in the DC group (odds ratio, 0.84; 95% CI, 0.58–1.23). However, wound-related complications were reported in 4 patients in the craniotomy group, whereas 17 were reported in the DC group, and surgical-site infections were reported in 5 patients in the craniotomy group and 10 in the DC group. The craniotomy group required additional procedures more often within 2 weeks, most of them being DC (9% of the craniotomy group had a secondary DC).

Concerning primary DC for mass lesions, there is a consensus that, after evacuating an ASDH, if the brain is bulging beyond the inner table of the skull intraoperatively, leaving the bone flap out is the best option. The RESCUE-ASDH trial results suggest that, if the bone flap can be replaced without compressing the brain, surgeons may consider doing so instead of performing a preemptive DC.^13^ A multicenter international prospective observational study (Collaborative European NeuroTrauma Effectiveness Research in Traumatic Brain Injury [CENTER-TBI]) enrolled 336 patients with ASDH requiring surgical evacuation. It concluded that DC and craniotomy result in similar functional outcomes.^25^ However, primary DC should be restricted to salvageable patients for whom replacement of the bone flap is not possible due to intraoperative brain swelling.^25^ Moreover, a hinge craniotomy (HC) might be an alternative solution.

## SECONDARY DC

Two landmark randomized controlled trials (RCTs), Decompressive Craniectomy in Diffuse Brain Injury (DECRA)^12^ and Randomized Evaluation of Surgery with Craniectomy for Uncontrollable Elevation of Intracranial Pressure (RESCUEicp),^24^ addressed the question of whether secondary DC as a neuroprotective or last-tier procedure, respectively, leads to better outcomes compared with medical management. Decompressive Craniectomy in Diffuse Brain Injury enrolled TBI patients who received tier 1 treatment with ICP higher than 20 mm Hg for 15 minutes over a 1-hour period within the first 72 hours of care (early), while RESCUEicp enrolled patients with ICP greater than 25 mm Hg for 1 to 12 hours refractory to 2 tiers of treatment within 10 days of admission (late).^12,24^ The current overall evidence from the two RCTs is summarized in Table 2.

**TABLE 2:** Summary of the Three Key Messages From the DECRA and RESCUEicp Trials

1. Early neuroprotective bifrontal DC for mild to moderate intracranial hypertension is not superior to medical management for patients with diffuse TBI^12^
2. Unilateral or bifrontal DC used as a last-tier therapy for patients with severe, sustained, and refractory posttraumatic intracranial hypertension leads to a substantial mortality reduction compared with medical management^24^
3. DC patients with traumatic intracranial hypertension are more likely to improve over time (6 to 24 mo) compared with patients in the standard medical treatment group^24,26^

In the DECRA trial, 155 patients were randomly assigned to bifrontotemporoparietal DC or medical management, with the primary outcome being the GOSE score at 6 months.^12^ Patients undergoing DC had less time with ICP above the treatment threshold (*p* < 0.001), fewer interventions for increased ICP (*p* < 0.02 for all comparisons), and fewer days in the intensive care unit (*p* < 0.001). However, the DC group had a worse GOSE score than those receiving medical therapy, with an odds ratio of 1.84 (95% CI, 1.05–3.24; *p* = 0.03) and a greater risk of unfavorable outcome (odds ratio, 2.21; 95% CI, 1.14–4.26; *p* = 0.02) (Table 1). However, at 12 months, the GOSE was no longer significantly worse in the DC group.^27^ Rates of death at 6 months were similar in the DC group (19%) and the standard-care group (18%). The number of deaths at 12 months was also similar, 21% in the DC versus 19% in the craniotomy group. Regarding covariates impacting outcomes, the DC group had a higher proportion of patients with bilateral unreactive pupils. Following post hoc adjustment for baseline pupil reactivity, there was no difference in unfavorable GOSE outcomes.

The RESCUEicp was an international, multicenter, parallel-group RCT that enrolled TBI patients between 10 and 65 years of age with refractory elevated ICP despite stage 1 and 2 treatments (Table 1).^24^ The surgical treatment was either a large unilateral frontotemporoparietal craniectomy (hemicraniectomy) or bifrontal craniectomy. The primary outcome measure was the GOSE at 6 months after randomization (Table 1). The baseline characteristics between the two populations were similar. At 6 months, the DC group had a significantly lower mortality rate than the medical group (26.9% vs. 49.9%). For every 100 patients treated with surgical rather than medical intent, there were 22 more survivors; of these 22 patients, 6 (27%) were in a vegetative state, 8 (36%) were categorized as having lesser severe disability, and 8 (36%) were categorized as having higher severe disability or better. In 2022, the 2-year follow-up data from the RESCUEicp trial concluded that, for every 100 individuals treated surgically, 21 additional patients survived at 24 months; 4 were in a vegetative state, 2 had lesser severe disability, 7 had higher severe disability, 5 had lesser moderate disability, and 3 had higher moderate disability.^26^ The DC group were more likely to recover over time, denoted by the significant differences in net improvement (≥1 grade) between 6 and 24 months (55 [30.0%] vs. 25 [14.0%]; χ^2^ = 13.27, *p* = 0.001).^26^

The updated 2020 BTF guidelines recommended that secondary DC be performed for late refractory ICP elevation to improve mortality and favorable outcomes but not for early refractory ICP elevation. When performed, a large frontotemporoparietal DC (15 cm in diameter) was recommended.^9,12^ The statement from an international consensus meeting suggests that ICP monitoring to aid decision making is warranted in combination with radiological and neurological findings, with the best candidate for secondary DC being a patient whom ICP elevation is the primary contributor to poor outcomes and in whom the primary injury is deemed appropriate for rehabilitation.^28^ However, it remains uncertain which patient subgroups might benefit more.^28^ Decompressive craniectomy was recommended as a tier 3 treatment option in the Seattle International Severe Traumatic Brain Injury Consensus Conference management algorithms.^29,30^

The overall data suggest that secondary DC has a role in the management of TBI but is not a panacea. The decision to offer this operation should be on a case-by-case basis.

## SURGICAL CONSIDERATIONS

### Size, Shape, and Dura

Surgical decision making for DC includes the size of the decompression, anatomical location, handling of the dura, use of adjuncts, and postoperative ICP monitoring. One multicenter and one single-center RCT evaluated the effects of the size of DC.^31,32^ Both studies compared unilateral frontotemporoparietal craniectomy standard trauma craniectomy (STC) with a bone flap size of 12 × 15 cm to a limited smaller (LC) temporoparietal craniectomy (8 × 6 cm). Frontotemporoparietal craniectomy was found to be associated with lower mortality (26.2%) compared with LC (35.1%; *p* < 0.05), with favorable GOS outcomes at 6 months in 39.8% of STC patients compared with 28.6% of LC patients (*p* = 0.05), and 12-month favorable outcomes of 56.8% and 32.4%, respectively (*p* = 0.035).^9,30,33–35^ Frontotemporoparietal craniectomy was associated with larger ICP reduction and lower rates of postoperative hematoma and subdural effusion. The 2020 BTF guidelines recommend a large rather than small STC DC (not less than 12 × 15 cm or 15 cm in diameter) for reducing mortality in patients with severe TBI.^9,33^ For both bifrontal and unilateral hemicraniectomy, the relative risks and benefits are an important gap in knowledge.^28^ A working hypothesis is that DC should be tailored to intracranial pathology in a personalized manner, that is, bifrontal DC might be better suited for patients with bifrontal/bitemporal contusions and bilateral brain edema and no midline shift. In contrast, a unilateral hemicraniectomy may benefit patients with lesions (extra-axial or parenchymal) predominantly in one hemisphere, with midline shift.^10,11,28,36^ Importantly, the skin incision should be larger than the intended size of the craniectomy, and the pinna should be avoided.^28^ The bone flap size should be large, and the dura should be widely opened.^28,36,37^ The optimal materials for duraplasty, the necessity of sutured expansile duroplasty, the method of bone flap storage, and the removal (or not) of bone overlying the superior sagittal sinus when performing bifrontal DC remain areas for further research.^28^ An RCT found no difference in sutured duraplasty versus nonsutured duraplasty in wound dehiscence, 10/69 versus 4/37, respectively (*p* = 0.766), or in surgical site infection, 6/69 versus 2/37, respectively (*p* = 0.710).^38^

### Decompressive Craniotomy

Regarding primary DC, the RESCUE-ASDH trial showed no overall benefit with DC versus craniotomy.^13^ However, following craniotomy, 18 of 228 patients required reoperation within 2 weeks. In addition, cranioplasty, which will be discussed further below, is needed to reconstruct the skull after primary or secondary DC. Consequently, an alternative surgical method to DC, termed HC or decompressive craniotomy (DCO), has been increasingly used in the last few years.^39,40^ Decompressive craniotomy allows the bone flap to “hinge” or “float” away from the surrounding skull, thus allowing some room for expansion of the swollen brain.^41,42^ There are more than 10 ways in which the concept of “HC” has been described and achieved, with varying degrees of success.^40^ Furthermore, according to an international survey, eight DCO techniques have been used, most loosely tying sutures to the bone flap.^40^ Most studies are retrospective single center and vary in intracranial pathologies, ICP monitoring, and postoperative outcomes, all of which yield only level III evidence.^39^ Across 60 countries (40 LMICs), DCO was carried out in 25% of cases, with the top three scenarios being ASDH with Glasgow Coma Scale (GCS) score of 9 to 12, ASDH with contusions and GCS score of 9 to 12, and ASDH with contusions and GCS score of 3 to 8.^40^ Decompressive craniotomy is a procedure most commonly carried out in LMICs.^40^ This technique is particularly important because it may simultaneously control ICP and obviate the need for cranioplasty. A second operation to reconstruct the cranium following DC, even with the use of autologous bone, could be financially detrimental to families in a country without health care coverage. Therefore, the technique's cost-effectiveness must be considered an equal priority to establishing evidence of its effectiveness (Table 3).^43^

**TABLE 3:** Key Outstanding Questions Regarding DCO

1. What is the best way to define DCO?
2. Is DCO a viable alternative to primary DC?
3. Is there a subgroup of patients that may benefit more from DCO?
4. Is DCO cost-effective compared with DC?

## CRANIOPLASTY

Following DC, the large skull defect leaves the brain unprotected and may contribute to jeopardized CSF dynamics. Cranial reconstruction (cranioplasty) is needed to restore the original skull contour. Computed tomography perfusion and ultrasonography have demonstrated improved cerebral blood flow following cranioplasty.^44,45^ Retrospective studies suggest that cranioplasty may aid neurological recovery.^44^

### Timing of Cranioplasty

The timing of cranioplasty following DC varies from weeks to months after injury.^43^ By convention, a cutoff of 90 days after DC has often been used to delineate “early” versus “late” cranioplasty.^46^ The UK cranioplasty registry reported 244 days median time to cranioplasty after DC.^47^ Three meta-analyses showed no difference in infection rates between early versus late cranioplasty.^46,48,49^ A systematic review of observational and case-controlled studies suggested that early cranioplasty may be associated with greater neurological improvement.^46^

### Complications of Cranioplasty

Cranioplasty can lead to significant intraoperative complications at every step of the procedure. It requires careful elevation of the scalp flap, separation of the dura and soft tissue, and preservation of the vasculature of the flap.^28^ Routine cranioplasty is associated with a higher infection rate than elective neurosurgical procedures.^50^ After DC, the ventricles may enlarge, and hydrocephalus may become prominent in 10% to 45% of cases.^46–49^ The risk of seizures ranges from 5% to 30%, and postoperative hematoma (predominantly epidural) can be as high as 10%.^46,51–53^

## DC IN CHILDREN

Traumatic brain injury is the leading cause of death in children older than 1 year.^54^ Survivors of pediatric TBI may experience cognitive and behavioral dysfunction, making it difficult for them to reintegrate into a social environment.

Only one RCT of DC in children has been performed.^27^ This pilot trial involved 27 children (median age, 120.9 months; range, 13.6–176.4 months) with head injuries who were randomly assigned to medical management alone or medical management plus bitemporal DC, which was performed at a median of 18.2 hours (range, 7.3–29.3 hours). Two of the 14 children (14%) in the control group were normal or had a mild disability after 6 months, compared with 7 (54%) of the 13 children in the decompression group. The authors also found that a DC for raised ICP in children can reduce ICP. The study design and execution had many limitations, including the fact that it was not adequately powered a priori, the quality of the evidence was low because of bias from the termination of the trial, the allocation method changed midway through the study, participant allocation was not concealed from the treatment team, the dura mater remained intact during the surgical procedure, and follow-up time was short. The Guidelines for the Management of Pediatric Severe Traumatic Brain Injury concluded that there is only level III evidence for the role of DC in ICP control.^17^ A multicenter prospective pediatric craniectomy and cranioplasty registry is currently enrolling patients.^55^

## THE USE OF DC IN RESOURCE-POOR ENVIRONMENTS AND MILITARY SETTINGS

Over recent decades, the incidence of global trauma has risen significantly, impacting mortality and morbidity rates in LMICs, where trauma remains a leading cause of death.^3,5^ Patients with severe TBI ideally require transfer to major trauma centers with specialized neurocritical care and neurosurgery. However, LMICs face delays in neurosurgical emergency care because of workforce shortages and the absence of formal prehospital systems. In addition, the lack of adequate infrastructure for postoperative care impedes the implementation of treatment protocols established in higher-income settings.

### Damage-Control Approaches in Neurotrauma

Patients face prolonged transfer times to specialized centers in noncapital cities, rural areas, and military front lines in LMICs. Damage-control in neurotrauma (DCNt) approaches have been developed as a response to such conditions. The DCNt aims to prevent the potentially lethal chain of expanding intracranial hematomas and cerebral edema following TBI, resulting in increased ICP and exacerbated secondary brain injury that may lead to herniation. Decompressive craniectomy can interrupt this cascade.^56^

The DCNt approach involves urgent neurosurgery with the goals of reducing secondary brain injury and stabilizing the patient for safe transfer. Principles include early use of hyperosmolar therapy, arrest of intracranial bleeding, and evacuation of intracranial hematomas. Further aims include limiting contamination of compound wounds through early surgical debridement, alleviating refractory intracranial hypertension, preventing herniation, decompressing the brainstem, and restoring anatomical continuity. A recent study by Brazilian neurosurgeons found no significant differences in patients with and without watertight dural closure after DC in a civilian setting. The study compared various parameters, including GOSE, with no significant disparities between the groups.^57^

Military conflicts have led to an increase in penetrating and blunt head trauma among military and civilian populations.^56^ Traditional frontline approaches have involved conservative measures and transfer to military hospitals for surgical intervention. However, recent conflicts have witnessed a paradigm shift toward early front-line DC, followed by overseas patient transfer.^58^ Experiences from military front lines, with associated risks of prolonged patient evacuation overseas, have contributed to the increased use of DCNt in low-resource civilian settings. Civilian patients without air transport may experience delayed transfers, resulting in worse clinical outcomes than military personnel receiving quicker transfers to nearby hospitals after initial surgery.^58^ Prolonged transportation for civilians may lead to significant cerebral edema and increased ICP, potentially leading to secondary brain injury. The DCNt approach allows for treatment without the need for ICP monitoring.^58^

## ETHICAL CONSIDERATIONS OF DC

Ethical considerations for DC have always revolved around the issue of increased survival versus significant disability and reduced overall quality of life.^59^ When secondary DC is needed for TBI, the RESCUEicp and DECRA trials provide evidence to aid discussions with family and relatives.^12,24^ In raw numbers, the RESCUEicp trial showed that, for every 100 individuals treated surgically, 21 additional patients survived at 24 months; 4 were in a vegetative state, 2 had lesser severe disability, 7 had higher severe disability, 5 had lesser moderate disability, and 3 had higher moderate disability.^29^

To address these issues, a multidisciplinary team-based approach is required.^59^ Access to care and resource allocation are among the considerations, focusing on disparities in socioeconomic status and the impact on outcomes for patients who have undergone DC. Therefore, the overall economic cost and societal burden of providing lifelong care are also factors to be considered.

We should also remember that what is accepted as a good long-term outcome for one patient may not be acceptable for another. The postoperative period of intense rehabilitation may not always lead to reintegration into society, and some families may not even have the resources to support this. Therefore, it is crucial to explicitly and openly discuss the surgical options, outline the advantages and disadvantages of each, and make joint decisions with families/relatives.

## CONCLUSION

Primary DC for ASDH leads to equivalent GOSE outcomes at 6 and 12 months as craniotomy. A relatively small subgroup of patients from the craniotomy group will subsequently require removal of the bone flap. Overall, we recommend replacing the bone flap in the absence of brain swelling. Importantly, patients undergoing DC may have more wound-associated complications.

Patients undergoing secondary DC as a last-tier therapy for refractory intracranial hypertension have reduced mortality and can improve over time compared with those who received standard medical management. Secondary DC should be considered on a case-by-case basis, in collaboration with the next of kin or other legal representative. Decompressive craniotomy is an alternative option when cranioplasty is not feasible or when the brain is neither bulging nor relaxed after primary evacuation. A high-quality trial is needed to refine the indications and optimal type of DCO.
